# Impact of Immunosuppressive Therapy on Lead Dislodgement After Cardiac Implantable Electronic Device Implantation

**DOI:** 10.1002/clc.24310

**Published:** 2024-06-18

**Authors:** Yasuhiro Matsuda, Masaharu Masuda, Mitsutoshi Asai, Shin Okamoto, Takayuki Ishihara, Kiyonori Nanto, Takuya Tsujimura, Yosuke Hata, Hiroyuki Uematsu, Naoko Higashino, Sho Nakao, Masaya Kusuda, Toshiaki Mano

**Affiliations:** ^1^ Kansai Rosai Hospital Cardiovascular Center Amagasaki Hyogo Japan

**Keywords:** cardiac electric implantable device implantation, immunosuppressive therapy, lead dislodgement

## Abstract

**Backgrounds:**

Lead dislodgement is a severe complication in cardiac implantable electronic device (CIED) implantation. Inflammation after CIED implantation results in the development of adhesions between lead and tissues, resulting in the lead becoming fixed in the body. In patients with immunosuppressive therapy, however, adhesion is inhibited by anti‐inflammatory effects. However, the association between lead dislodgement and immunosuppressive therapy has not been clarified. The purpose of this study was to investigate the association between lead dislodgement and immunosuppressive therapy.

**Hypothesis:**

We hypothesized that lead dislodgement more frequently occur in patients with immunosuppressive therapy than those without.

**Methods:**

In total, 651 consecutive patients who underwent CIED implantation or lead addition (age, 76 ± 11 years; and males, 374 [58%], high voltage device, 121 [19%], lead addition 23 [4%]) were retrospectively enrolled. Immunosuppressive therapy was with regular steroids or immunosuppressants. Lead placement was guided by fluoroscopy, and active fixation leads were used. Restraint of the upper limb by chest tape was performed for 1 week after the procedure. Lead dislodgement was defined as a change in lead position and/or lead failure requiring reoperation.

**Results:**

Twenty (3.1%) patients received immunosuppressive therapy. Among these, 15 (2.3%) patients regularly took steroids and 8 (1.2%) took immunosuppressants. Lead dislodgement occurred in 10 (1.5%) patients. Lead dislodgement was more frequent in patients with immunosuppressive therapy than in those without (3 [15%] vs. 7 [1%], *p* = 0.003).

**Conclusion:**

In patients with CIED implantation or lead addition, lead dislodgement is more frequent in patients with immunosuppressive therapy than in those without.

## Graphical abstract

In patients undergoing cardiac implantable electronic device (CIED) implantation or lead addition, lead dislodgement after the procedure was more frequent in patients with immunosuppressive therapy, namely regular use of steroids or immunosuppressants, than those without.

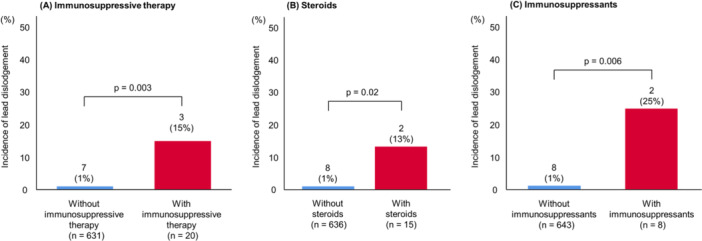

## 1 Introduction

Recent developments in nonpharmacotherapy for arrhythmia have been remarkable [1]. Of particularly note, cardiac implantable electric device (CIED) implantation has been established as a beneficial therapy for bradycardia, sudden death by ventricular tachyarrhythmia and impaired cardiac function. Nevertheless, one severe complication in CIED implantation is lead dislodgement [1, 2]. After CIED implantation, adhesions between lead and tissues begin to form due to inflammation and the fibrinolytic system [3, 4, 5], resulting in the fixation of the lead in the body. In patients receiving immunosuppressive therapy, however, such as steroids or immunosuppressants, adhesion is inhibited by anti‐inflammatory effects, and lead dislodgement appears to have accordingly increased [3, 6, 7]. To our knowledge, however, the association between lead dislodgement and immunosuppressive therapy have not been formally investigated. Here, we investigated the impact of immunosuppressive therapy on lead dislodgement.

## 2 Methods

### 2.1 Study Design and Patients

The study was conducted under a single‐center, retrospective observational design. Between January 2014 to December 2022, 651 consecutive patients who underwent CIED with intravenous leads, including pacemaker, defibrillator, and cardiac resynchronization devices, implantation, or lead addition, were retrospectively enrolled.

Data on comorbidities, medication, laboratory findings, and echocardiography before the procedure were collected. Immunosuppressive therapy was defined as regular use of steroids or immunosuppressants. Steroid dosage was calculated as the equivalent dose of prednisolone [8].

This study complied with the Declaration of Helsinki. Written informed consent for the CIED implantation procedure and participation in the study was obtained from all patients, and the protocol of this study was approved by the Kansai Rosai Hospital Institutional Review Board (Reference number: 22D104g).

### 2.2 CIED Implantation Procedure

CIED implantation was performed under local anesthesia in the angiography room or operating room of our hospital. In general, immunosuppressive therapy and antiplatelet agents were continued in the periprocedural period. Oral anticoagulants were stopped on the day of the procedure, and reinitiated after hemostasis was confirmed. As an antibiotic, cefamezin or ampicillin/sulbactam was administered 1 h before the procedure and continued for up to 4 days.

Extrathoracic axillar puncture was performed under venography guidance. A pocket was created in the prepectoral subcutaneous space, and leads were emplaced. The pocket was flushed with saline, and leads were connected to a generator. Finally, the subcutaneous tissue and skin were closed with absorbable sutures.

### 2.3 Details of Lead Placement

Lead placement was performed under fluoroscopic guidance in the antero‐posteria, right anterior oblique, and left anterior oblique views. Active fixation leads were used in the right atrium and right ventricle. Both passive fixation leads and active fixation leads were used as left ventricular lead. In general, sites of lead placement were right atrial appendage for the right atrial lead, right ventricular apex or right ventricular septum for the right ventricular lead, and lateral vein of the coronary sinus for the left ventricular lead. The presence of injury current at the time of fixation was at the operator's judgment [9]. The left ventricular lead was emplaced using a guiding catheter and 0.014‐inch guidewire.

For the right atrial lead and right ventricle lead, pacing by maximum output was performed and the absence of phrenic nerve stimulation was confirmed. After lead placement, we also confirmed that there was no lead dislodgement or pacing failure by asking the patient to take a deep breath and to cough.

The target CIED lead profile as follows: (1) amplitude was ≥1.0 mV for the right atrial lead and ≥5.0 mV for the right ventricular lead or left ventricular lead; (2) pacing threshold at a 0.4‐ms pulse duration was ≤1.5 V for the right atrial lead, ≤1.0 V for the right ventricular lead, and ≤2.0 V for the left ventricular lead; and (3) lead impedance was within the range of 300–1000 Ω.

After lead placement, leads were fixed to the pectoral muscle by two or three stitches of nonabsorbable suture using anchoring sleeves. Thereafter, in general, nonabsorbable sutures were used between the generator and pectoral muscle, with one stitch for pacemakers and two stitches for other types of CIED.

Chest X‐ray was performed just after the procedure. Chest tape was used to restrain the upper limb. Ambulation was permitted the next morning following the procedure. Pacemaker check, electrocardiogram, and chest X‐ray were performed at 1 week after the procedure. Chest tape was removed at the same time.

Similarly to previous studies, lead dislodgement was defined as a change in lead position and/or a lead failure requiring reoperation [10, 11]. Other complications were also defined based on previous studies [10, 11].

Follow‐up of the wound was performed at 1 week after discharge. Pacemaker check, 12‐lead electrocardiogram, and chest X‐ray were performed at 1 month after discharge. In general, pacemakers were checked and electrocardiograms were conducted every 6–12 months thereafter.

### 2.4 Statistical Analysis

Categorical data are presented as absolute values and percentages, and continuous data as the mean ± standard deviation or median (interquartile range). Tests for significance were conducted using the unpaired Student's *t*‐test or Mann–Whitney *U*‐test for continuous variables, and the *χ* ^2^ test or Fisher's exact test for categorical variables. Associations between immunosuppressive therapy and lead dislodgement were analyzed using unadjusted and adjusted logistic regression analyses. In the adjusted analyses, immunosuppressive therapy was adjusted for variables with a *p* value ≤ 0.05 in the unadjusted analysis. All analyses were performed using commercial software (SPSS version 25™, SPSS, Inc., Chicago IL, USA).

## 3 Results

### 3.1 Patient Characteristics and Procedural Characteristics

CIED implantation (*n* = 628, [96%]) and lead addition (*n* = 23, [4%]) were successfully performed in all patients, consisting of a pacemaker in 530 (81%); implantable cardioverter defibrillator in 72 (11%); cardiac resynchronization therapy pacemaker in 17 (3%); and cardiac resynchronization therapy defibrillator in 32 (5%). Baseline heart disease in pacemaker patients was sick sinus syndrome in 188 (35%), atrioventricular block in 321 (61%), and atrial fibrillation with bradycardia in 21 (4%). Device vendors were Medtronic (224 [34%], Minneapolis MN, USA), Abbott (208 [32%], Abbott Park IL, USA), Nihon Kohden (76 [12%], Tokyo, Japan), Boston Scientific (56 [9%], Marlborough MA, USA), Sorin (54 [8%], Mirandola, Italy), and Biotronik (33 [5%], Berlin, Germany). Detailed information on CIED leads were described in Table S1. All CIED leads were steroid eluting. Additonally, patient characteristics are shown in Table 1, and procedural characteristics in Table 2.

**Table 1:** Patient characteristics.

Variable	All (*n* = 651)	Without lead dislodgement (*n* = 641)	With lead dislodgement (*n* = 10)	*p*	Without immunosuppressive therapy (*n* = 631)	With immunosuppressive therapy (*n* = 20)	*p*
Age, years	76 ± 11	76 ± 11	76 ± 10	0.98	76 ± 11	75 ± 12	0.67
Male, *n* (%)	374 (58)	371 (58)	3 (30)	0.11	363 (58)	11 (55)	0.82
Body mass index (kg/m^2^)	23 ± 4	23 ± 4	23 ± 4	0.99	23 ± 4	21 ± 3	0.006
Temporary cardiac pacing, *n* (%)	188 (29)	183 (29)	5 (50)	0.16	178 (28)	10 (50)	0.03
Hypertension, *n* (%)	382 (59)	380 (59)	2 (20)	0.02	372 (59)	10 (50)	0.42
Diabetes mellitus, *n* (%)	168 (26)	165 (26)	3 (30)	0.72	162 (26)	6 (30)	0.66
Hemodialysis, *n* (%)	48 (7)	47 (7)	1 (10)	0.54	46 (7)	2 (10)	0.65
Stroke, *n* (%)	48 (7)	47 (7)	1 (10)	0.54	48 (8)	0 (0)	0.39
Atrial tachyarrhythmia, *n* (%)	202 (31)	200 (31)	2 (20)	0.73	199 (32)	3 (15)	0.14
Chronic obstructive pulmonary disease, *n* (%)	13 (2)	13 (2)	0 (0)	1.00	13 (2)	0 (0)	1.00
Past history of device erosion, *n* (%)	8 (1)	8 (1)	0 (0)	1.00	8 (1)	0 (0)	1.00
Coronary artery disease, *n* (%)	203 (31)	198 (31)	5 (50)	0.30	195 (31)	8 (40)	0.39
Open heart surgery, *n* (%)	73 (11)	72 (11)	1 (10)	1.00	72 (11)	1 (5)	0.37
Antiplatelet agent, *n* (%)	225 (35)	223 (35)	2 (20)	0.51	219 (35)	6 (30)	0.66
Oral anticoagulant, *n* (%)	171 (26)	170 (27)	1 (10)	0.47	169 (27)	2 (10)	0.12
NSAIDs, *n* (%)	31 (5)	29 (5)	2 (20)	0.08	27 (4)	4 (20)	0.01
Hemoglobin (g/dL)	12 ± 2	12 ± 2	11 ± 2	0.16	12 ± 2	11 ± 2	0.08
Brain natriuretic peptide (pg/mL)	188 (80–544)	188 (79–542)	450 (124–817)	0.33	188 (82–541)	478 (20–1243)	0.80
NT‐Pro BNP (pg/mL)	832 (288–2776)	838 (292–2824)	299 (88–n/a)	0.32	811 (280–2776)	1838 (342–3126)	0.46
eGFR (mL/min/1.73 m^2^)	51 ± 24	51 ± 24	46 ± 27	0.51	51 ± 24	49 ± 26	0.77
Left ventricular ejection fraction (%)	59 ± 16	59 ± 16	60 ± 15	0.86	59 ± 16	58 ± 15	0.69

*Note:* Values indicate mean ± standard deviation or median (1st quartile–3rd quartile).

Abbreviations: eGFR: estimated glomerular filtration rate, NSAIDs: nonsteroidal anti‐inflammatory drugs, NT‐Pro BNP: N‐terminal pro‐brain natriuretic peptide.

**Table 2:** Procedural characteristics.

Variable	All (*n* = 651)	Without lead dislodgement (*n* = 641)	With lead dislodgement (*n* = 10)	*p*	Without immunosuppressive therapy (*n* = 631)	With immunosuppressive therapy (*n* = 20)	*p*
Lead addition, *n* (%)	23 (4)	20 (3)	3 (30)	0.004	21 (3)	2 (10)	0.15
High voltage device, *n* (%)	121 (19)	118 (18)	3 (30)	0.41	115 (18)	6 (30)	0.18
Left ventricular lead implantation, *n* (%)	48 (7)	47 (7)	1 (10)	0.54	46 (7)	2 (10)	0.65
Procedure from the right side, *n* (%)	68 (10)	66 (10)	2 (20)	0.28	65 (10)	3 (15)	0.46
Dual chamber pacemaker, *n* (%)	583 (90)	573 (89)	10 (100)	0.61	565 (90)	18 (90)	1.00
Procedural time (min)	119 ± 45	119 ± 45	137 ± 59	0.21	119 ± 44	132 ± 57	0.19
Right atrial wave amplitude (mV)	2.5 ± 1.3	2.5 ± 1.3	1.8 ± 0.6	0.13	2.6 ± 1.4	2.4 ± 1.1	0.58
Right atrial pacing threshold (V)	1.0 ± 0.5	1.0 ± 0.5	1.0 ± 0.3	0.77	1.0 ± 0.5	1.1 ± 0.4	0.21
Right atrial lead impedance (Ω)	526 ± 111	526 ± 110	515 ± 137	0.78	527 ± 110	487 ± 107	0.15
Right ventricular wave amplitude (mV)	9.1 ± 4.4	9.1 ± 4.4	7.9 ± 2.9	0.47	9.1 ± 4.5	8.4 ± 3.2	0.53
Right ventricular pacing threshold (V)	0.6 ± 0.2	0.6 ± 0.2	0.6 ± 0.1	0.60	0.6 ± 0.2	0.5 ± 0.1	0.02
Right ventricular lead impedance (Ω)	636 ± 145	636 ± 144	622 ± 224	0.80	638 ± 145	571 ± 139	0.06
Left ventricular wave amplitude (mV)	9.2 ± 6.1	9.2 ± 6.1	—	—	9.5 ± 6.0	1.0	0.18
Left ventricular pacing threshold (V)	1.3 ± 0.9	1.3 ± 0.9	—	—	1.3 ± 0.9	1.6 ± 0.2	0.58
Left ventricular lead impedance (Ω)	698 ± 225	698 ± 225	—	—	705 ± 229	564 ± 93	0.40

*Note:* All values indicate mean ± standard deviation unless otherwise indicated.

### 3.2 Lead Dislodgement and Immunosuppressive Therapy

**Figure 1:**
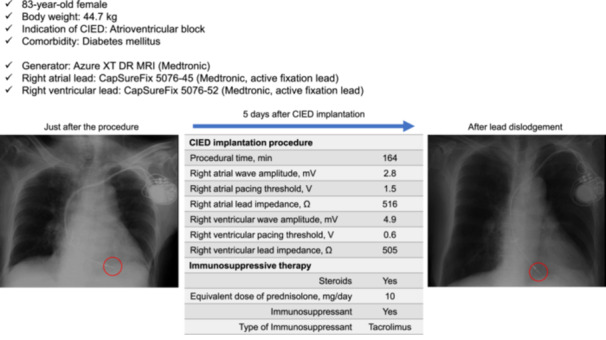
Representative case of lead dislodgement in a patient receiving immunosuppressive therapy. In this patient, right ventricular lead dislodgement occurred 5 days after pacemaker implantation. After reoperation, superficial site infection also occurred and close observation with intravenous antibiotics was needed. CIED, cardiac implantable electric device.

Lead dislodgement occurred in 10 (1.5%) patients. The details of lead dislodgement are shown in Table S2, and a representative case is shown in Figure 1. Median time to lead dislodgement from the procedure was 6 (2–12) days. In patients with lead dislodgement, 3 (30%) patients were on remote monitoring. Symptoms or CIED malfunctions occurred in 7 (70%) patients, and lead dislodgements were detected by chest X‐ray and/or pacemaker check. Lead dislodgements in 2 (20%) patients were detected after discharge. The details of symptoms and CIED malfunctions were phrenic nerve stimulation (two patients), dyspnea (one patient), fatigue (one patient), bradycardia (one patient), sensing failure (one patient), and pacing failure (one patient). There was no significant difference in years of operator's experience between patients with lead dislodgement and those without (9 [6–11] vs. 9 [7–12] years, respectively, *p* = 0.66). Regarding left ventricular lead, there was no significant difference in lead dislodgement between passive fixation lead and active fixation lead (1 [3%] vs. 0 [0%], respectively, *p* = 1.00).

Twenty (3.1%) patients received immunosuppressive therapy. Compared with those without, patients with immunosuppressive therapy had a lower body mass index; higher prevalence of nonsteroidal anti‐inflammatory drug use (Table 1); and lower pacing threshold in the right ventricle lead (Table 2). Indications for immunosuppressive therapy were rheumatoid arthritis (nine patients), sarcoidosis (two patients), polymyalgia rheumatica (one patient), malignant lymphoma (one patient), adrenal insufficiency (one patient), anti‐neutrophil cytoplasmic antibody‐associated vasculitis (one patient), autoimmune pancreatitis (one patient), uterine cancer (one patient), eosinophilia (one patient), and unknown (two patients).

Among patients who received immunosuppressive therapy, 15 (2.3%) patients regularly took steroids. The equivalent dose of prednisolone was 3 (2–5) mg/day. Eight (1.2%) patients took immunosuppressants. Types of immunosuppressant were methotrexate (five patients), bucillamine (two patients), and tacrolimus, cyclophosphamide, salazosulfapyridine, etanercept, and mizoribine (one patient each).

**Figure 2:**
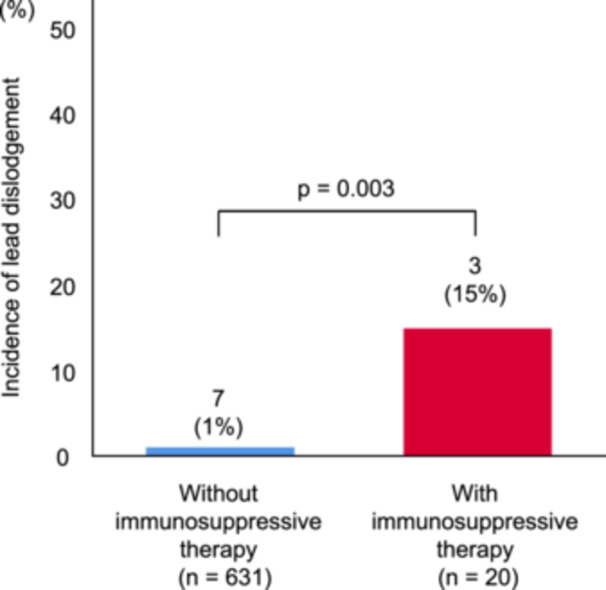
Incidence of lead dislodgement and immunosuppressive therapy. Lead dislodgement after the procedure was more frequent in patients with immunosuppressive therapy than those without.

Lead dislodgement occurred more frequently in patients with immunosuppressive therapy than in those without (Figure 2). The details of immunosuppressive therapy in patients with lead dislodgement are shown in Table S2. The severe complication rate related to the procedure is shown in Table S3, revealing no significant difference between those with and without immunosuppressive therapy except for lead dislodgement. In subgroup analysis, lead dislodgement was more frequent in patients with regular steroid usage than in those without (Figure S1A). Similarly, lead dislodgement was more frequent in patients with immunosuppressant usage than in those without (Figure S1B).

Although 24 (4%) patients received steroid premedication to prevent contrast allergy, there was no significant difference in the incidence of lead dislodgement between those receiving and not receiving premedication steroids (1 [4%] vs. 9 [1%], respectively, *p* = 0.32).

Patients with lead dislodgement had a lower prevalence of hypertension than those without (Table 1), and lead dislodgement was more frequent in patients with lead addition than those with CIED implantation (Table 2). After adjustment for hypertension, lead addition or nonsteroidal anti‐inflammatory drugs, immunosuppressive therapy was an independent predictor of lead dislodgement (Table S4).

## 4 Discussion

In this retrospective observational study of the association between immunosuppressive therapy and incidence of lead dislodgement among 651 patients undergoing CIED implantation or lead addition, we found: (1) of 651 patients, lead dislodgement occurred in 10 (1.5%) patients, while 20 (3.1%) patients received immunosuppressive therapy, namely regular use of steroids or immunosuppressants; (2) lead dislodgement was more frequent in patients with immunosuppressive therapy than in those without; and (3) after adjustment for other predictors of lead dislodgement, immunosuppressive therapy was an independent predictor of lead dislodgement. To our knowledge, this is the first clinical study to investigate the association of immunosuppressive therapy with lead dislodgement following CIED implantation or lead addition.

### 4.1 Lead Adhesion After CIED Implantation and Immunosuppressive Therapy

After CIED implantation, fibrous sheath formation around leads initially occurred at the tricuspid valve or border zone between the superior vena cava and right atrium [2]. The fibrous sheath then spread over the whole lead [2, 5]. Fibrous sheath formation is triggered by thrombus around the lead and/or endothelial injury at the surface of tissue [3, 4, 5]. Following this, neutrophils, monocytes, and macrophages infiltrate the tissue as an inflammatory reaction against foreign bodies, from which the formation of connective tissue proceeds [3, 4, 5].

In autoimmune disease, cell adhesion molecules such as E‐selectin, intercellular adhesion molecule‐1, and vascular cell adhesion molecule‐1 are activated [12, 13]. In the clinical setting, a previous study saw no significant difference in the number of postoperative days to wound healing between patients with and without rheumatoid arthritis [14]. Hence, auto‐immune disease itself does not seem to inhibit lead adhesion. However, steroids and immunosuppressants suppress the activity of leukocytes and inhibit the expression of cell adhesion molecules [12, 15, 16]. Formation of connective tissue is thereby delayed, and lead dislodgement may thereby increase. More than half of the lead dislodgements occurred within 1 week in our study. Even in cases occurring just after the procedure, inhibition of connective tissue formation by immunosuppressive therapy may affect lead dislodgement, because lead adhesion to vessels and myocardium begins within 24 h after CIED implantation [17].

In our study, steroid dose was low in patients with regular steroid use. Lead dislodgement nevertheless increased because steroids suppress the immune system even low doses [18].

### 4.2 Myocardial Vulnerability and Lead Dislodgement

In addition to inhibition of lead adhesion, another cause of lead dislodgement may be myocardial vulnerability.

Therefore vulnerable myocardium easily tear, dislodgement by lead tension after CIED implantation may increase [19]. Some autoimmune diseases, for which immunosuppressive therapy is indicated, cause myocardial injury, including inflammation, fibrosis, and necrosis [20, 21]. Long‐term steroids exposure induce myocardial fibrosis, and thus immunosuppressive therapy also weakens myocardium [22]. In our study, we saw no significant difference in the incidence of lead dislodgement between patients with and without single steroid premedication. This result might imply that one of the cause of lead dislodgement is myocardial vulnerability due to long‐term steroid therapy, as well as temporary immunosuppression.

### 4.3 Other Predictors of Lead Dislodgement

Hypertension was a negative predictor of lead dislodgement in our study, while lead addition was a positive predictor.

In patients with hypertension, collagen and macrophages in the vascular wall increase due to inflammation. This may induce lead adhesion and consequently decrease lead dislodgement [23]. Lead placement in the lead addition procedure is performed by avoiding already existing leads, and the sites of lead placement are limited. On this basis, lead dislodgement might be frequent.

Although nonsteroidal anti‐inflammatory drugs did not predict lead dislodgement in adjusted logistic regression analysis, lead dislodgement tended to occur in patients who received nonsteroidal anti‐inflammatory drugs than those without. As well as immunosuppressive therapy, nonsteroidal anti‐inflammatory drugs have an anti‐inflammatory effect and formation of connective tissue [24, 25]. However, steroids have a greater anti‐inflammatory effect than nonsteroidal anti‐inflammatory drugs [25], thus there may be no significant difference in lead dislodgement between patients who received nonsteroidal anti‐inflammatory drugs than those without.

### 4.4 Immunosuppressive Therapy and Infection

Reoperation and immunosuppressive therapy itself are risk factors of CIED infection [26, 27, 28]. However, in our study, there was no significant difference in infection between patients with immunosuppressive therapy and those without. In patients with immunosuppressive therapy and/or reoperation for lead dislodgement, operators might consciously try to preventive measures of CIED infection, such as close observation of wounds, long‐term antibiotic administration, and long compression of wounds to avoid hematoma which caused CIED infection [28].

### 4.5 Clinical Implications

This study has several potential clinical benefits. Patients receiving immunosuppressive therapy require close observation after CIED implantation. These patients should also avoid movement which induces lead dislodgement, such as excessive elevation of the upper limb. Additionally, more careful implantation procedures, such as confirmation of injury current, lead traction testing and large lead deflection, might also be considered.

### 4.6 Limitations

Several limitations of our study warrant mention. First, we were unable to assess mild dislodgement that did not require reoperation. Second, even though we attempted to standardize CIED implantation procedures to the extent possible, strategies and skills of the procedure might have varied among operators. For example, already‐known predictors of lead dislodgement, such as female, obesity, did not predict lead dislodgement in our study [29, 30]. In procedures for these patients, operators might have consciously set large lead deflection and firm affixation to the pectoral muscle to prevent lead dislodgement. Third, because this study used a single‐center and retrospective design, differences in patient characteristics and procedural characteristics were present, and some complication might be overlooked in spite of routine follow‐up. In addition, because not every patient was on remote monitoring in our study, lead dislodgement might detected later than the actual timing of lead dislodgement. Fourth, although a variety type of active fixation leads, including delivery catheter guided leads or active fixation left ventricular leads, were enrolled in our study, passive fixation leads were not used for right atrium leads and right ventricle leads. Therefore it might be insufficient to evaluate the association between immunosuppressive therapy and lead dislodgement by subtypes of leads. Finally, the number of lead dislodgement (10 [1.5%]) and patients who received immunosuppressive therapy (20 [3.1%]) were small in this study although the numbers were similar to those seen in previous studies (the incidence of lead dislodgement: 1.7%, and the rate of immunosuppressive therapy: 1.9%) [30, 31]. Therefore, sampling bias or selection bias can occur, and accordingly may affect the results of statistical analysis in this study.

## 5 Conclusions

In patients with CIED implantation or lead addition, lead dislodgement is more frequent in patients with immunosuppressive therapy than in those without. A careful strategy for lead placement and close observation after the procedure may be needed in patients who receive immunosuppressive therapy.

## Disclosure

The authors have nothing to report.

## Ethics Statement

Kansai Rosai Hospital Institutional Review Board. Reference number: 22D104g.

## Conflicts of Interest

The authors declare no conflicts of interest.

## Supporting information


**FIGURE S1 Incidence of lead dislodgement and regular steroid or immunosuppressant use. (A)** Lead dislodgement was more frequent in patients with regular steroid use than those without. (B) Lead dislodgement was more frequent in patients with immunosuppressant use than those without.

Supporting information.

Supporting information.

Supporting information.

Supporting information.

## Data Availability

The authors have nothing to report.
