# Supplementary figures and images for: Impact of Immunosuppressive Therapy on Lead Dislodgement After Cardiac Implantable Electronic Device Implantation
Source: Clin Cardiol. 2024 Jun 18;47(6):e24310. doi: 10.1002/clc.24310 (PMC11184469; doi:10.1002/clc.24310)

**(A) Steroids**

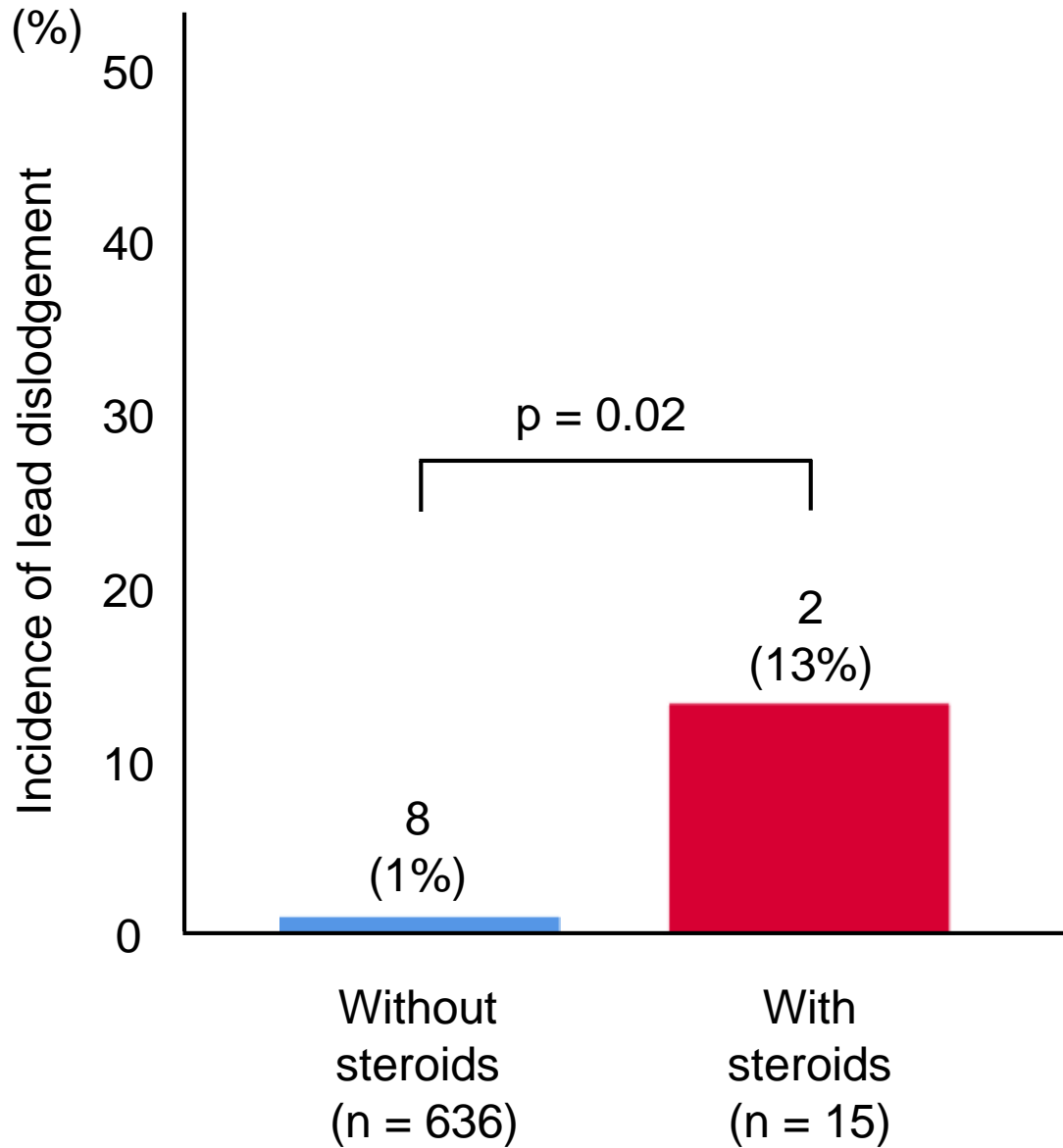

**(B) Immunosuppressants**

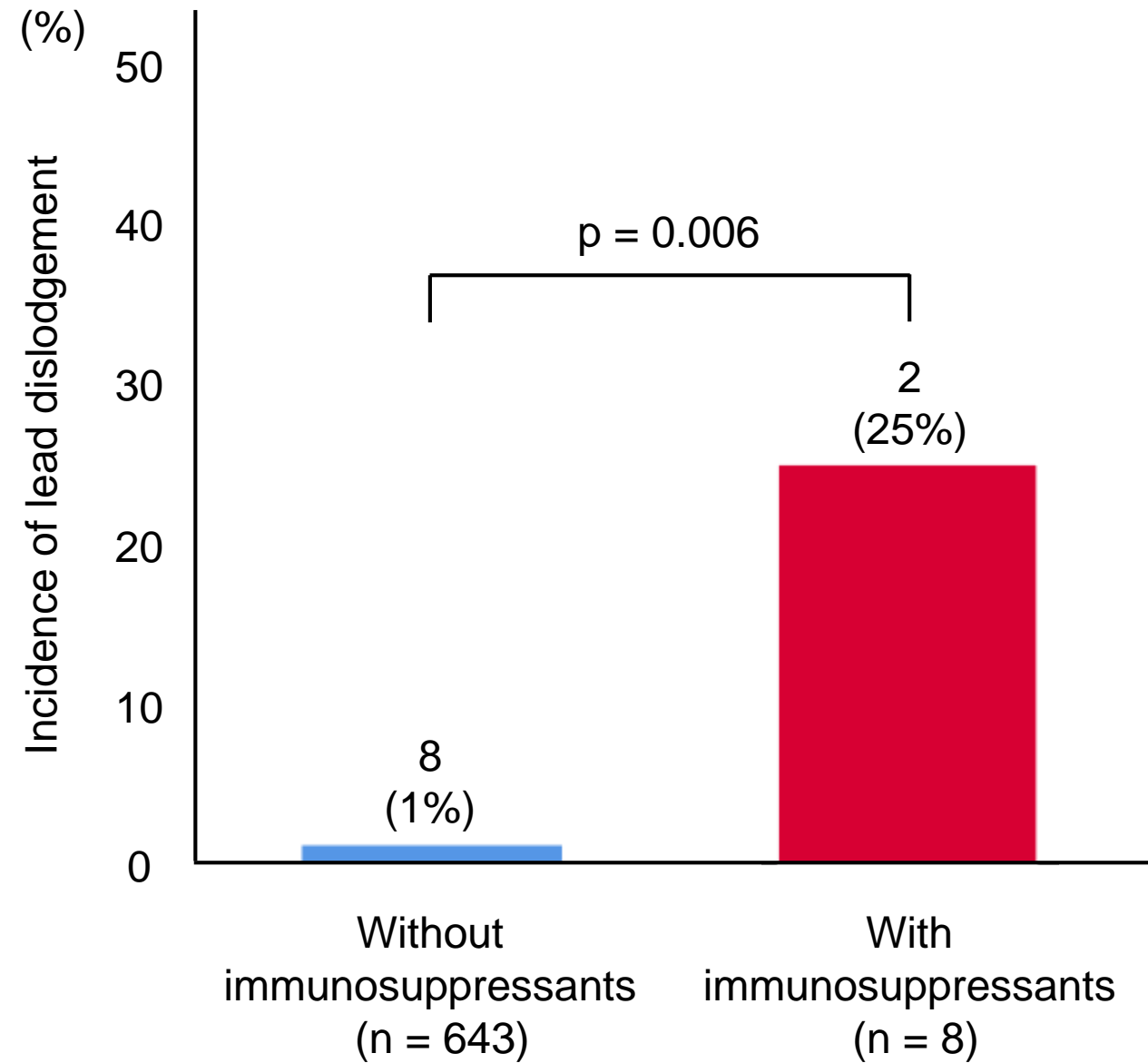

Supplement: Supplementary file 1 — FIGURE S1 Incidence of lead dislodgement and regular steroid or immunosuppressant use. (A) Lead dislodgement was more frequent in patients with regular steroid use than those without. (B) Lead dislodgement was more frequent in patients with immunosuppressant use than those without. [file CLC-47-e24310-s002.pdf]
