# Supplementary material for: Impact of Immunosuppressive Therapy on Lead Dislodgement After Cardiac Implantable Electronic Device Implantation
Source: Clin Cardiol. 2024 Jun 18;47(6):e24310. doi: 10.1002/clc.24310 (PMC11184469; doi:10.1002/clc.24310)
Supplement: Supplementary file 2 — Supporting information. [file CLC-47-e24310-s005.docx]

**Supplementary Table 1. Models of CIED leads**

| **Variable** | **All**  **n = 651** |
| --- | --- |
| Right atrial leads (n = 579) |  |
| Abbott Tendril (2088TC, LPA1200M), n (%) | 245 (42) |
| Abbott OptiSense, n (%) | 1 (0.2) |
| Biotronik Solia S, n (%) | 27 (5) |
| Boston Scientific INGEVITY (7740, 7741, 7742), n (%) | 49 (9) |
| Boston Scientific INGEVITY+ (7840), n (%) | 1 (0.2) |
| Boston Scientific FINE LINE II (4469), n (%) | 1 (0.2) |
| Oscor PY2, n (%) | 2 (0.3) |
| Sorin BEFLEX (RF45D, VEGA R), n (%) | 46 (8) |
| Medtronic CapsureFix (5076, 5086), n (%) | 205 (35) |
| Medtronic SelectSecure (3830), n (%) | 2 (0.3) |
| Right ventricular leads (n = 633) |  |
| Abbott Tendril (2088TC, LPA1200M), n (%) | 262 (41) |
| Abbott Durata (7120Q, 7122Q), n (%) | 18 (3) |
| Biotronik Solia S, n (%) | 3 (0.5) |
| Biotronik Plexa, n (%) | 14 (2) |
| Biotronik Protego, n (%) | 13 (2) |
| Boston Scientific INGEVITY (7741, 7742, 7841), n (%) | 47 (7) |
| Boston Scientific FINE LINE II (4470), n (%) | 1 (0.2) |
| Boston Scientific RELIANCE 4-FRONT, n (%) | 6 (0.9) |
| Oscor PY2, n (%) | 2 (0.3) |
| Sorin BEFLEX (RF45D, RF46D, VEGA R), n (%) | 50 (8) |
| Sorin INVICTA, n (%) | 1 (0.2) |
| Medtronic CapsureFix (5076, 5086), n (%) | 169 (27) |
| Medtronic SelectSecure (3830), n (%) | 4 (0.6) |
| Medtronic Sprint Quattro (6935, 6947), n (%) | 43 (7) |
| Left ventricular leads (n = 48) |  |
| Abbott Quartet (1456Q, 1458Q), n (%) | 17 (35) |
| Biotronik Sentus, n (%) | 7 (15) |
| Boston Scientific Acuity X4 (4672, 4678), n (%) | 4 (8) |
| Medtronic Attain Performa (4298, 4598), n (%) | 8 (17) |
| Medtronic Attain Ability (4296, 4396), n (%) | 2 (4) |
| Medtronic Attain Stability Quad, n (%) | 10 (21) |

CIED: Cardiac implantable electronic device
