# Supplementary material for: Impact of Immunosuppressive Therapy on Lead Dislodgement After Cardiac Implantable Electronic Device Implantation
Source: Clin Cardiol. 2024 Jun 18;47(6):e24310. doi: 10.1002/clc.24310 (PMC11184469; doi:10.1002/clc.24310)
Supplement: Supplementary file 3 — Supporting information. [file CLC-47-e24310-s003.docx]

**Supplementary Table 2. Details of lead dislodgement**

| **No.** | **Age,**  **years** | **Gender** | **Lead**  **addition** | **Type of CIED** | **Type of dislodged lead^†^** | **Time to lead dislodgement**  **from the procedure, days** | **Years of operator’s experience, years** | **Immunosuppressive therapy** | **Steroids** | **Equivalent**  **dose of prednisolone, mg/day** | **Immunosuppressant** | **Type of Immunosuppressant** |
| --- | --- | --- | --- | --- | --- | --- | --- | --- | --- | --- | --- | --- |
| 1 | 84 | Female | No | PM | RA,RV | 2 | 9 | No | No | - | No | - |
| 2 | 84 | Female | No | PM | RV | 64 | 5 | No | No | - | No | - |
| 3 | 62 | Male | No | ICD | RA | 7 | 12 | No | No | - | No | - |
| 4 | 84 | Female | No | PM | RV | 13 | 9 | No | No | - | No | - |
| 5 | 83 | Female | No | PM | RV | 5 | 6 | Yes | Yes | 10 | Yes | Tacrolimus |
| 6 | 82 | Female | No | PM | RV | 2 | 4 | No | No | - | No | - |
| 7 | 69 | Male | Yes | PM | RA | 2 | 8 | Yes | No | - | Yes | MTX,BUC,ETN |
| 8 | 74 | Male | Yes | CRT-D | LV | 2 | 8 | No | No | - | No | - |
| 9 | 55 | Female | Yes | ICD | RV | 7 | 10 | Yes | Yes | 5 | No | - |
| 10 | 81 | Female | No | PM | RA,RV | 12 | 17 | No | No | - | No | - |

CIED: cardiac implantable electronic device, PM: Pacemaker, ICD: Implantable cardioverter defibrillator, CRT-D: Cardiac resynchronization therapy defibrillator,

RA: Right atrial lead, RV: Right ventricular lead, LV: Left ventricular lead. MTX: Methotrexate, BUC: Bucillamine, ETN: Etanercept

^†^Left ventricular lead in patient No. 8 was a passive fixation lead. Other leads were active fixation leads.
