# Supplementary material for: Impact of Immunosuppressive Therapy on Lead Dislodgement After Cardiac Implantable Electronic Device Implantation
Source: Clin Cardiol. 2024 Jun 18;47(6):e24310. doi: 10.1002/clc.24310 (PMC11184469; doi:10.1002/clc.24310)
Supplement: Supplementary file 4 — Supporting information. [file CLC-47-e24310-s004.docx]

**Supplementary Table 3. Severe complications related to the procedure**

| **Variable** | **All**  **n = 651** | **Without immunosuppressive therapy**  **n = 631** | **With immunosuppressive therapy**  **n = 20** | **p** |
| --- | --- | --- | --- | --- |
| All, n (%) | 54 (8) | 50 (8) | 4 (20) | 0.08 |
| Lead dislodgement, n (%) | 10 (2) | 7 (1) | 3 (15) | 0.003 |
| Cardiac arrest within 24 hours of the procedure, n (%) | 1 (0.2) | 1 (0.2) | 0 (0) | 1.00 |
| Death within 30 days related to the procedure, n (%) | 2 (0.3) | 2 (0.3) | 0 (0) | 1.00 |
| Hematoma, n (%) | 9 (1) | 9 (1) | 0 (0) | 1.00 |
| Cardiac tamponade, n (%) | 10 (2) | 10 (2) | 0 (0) | 1.00 |
| Pneumothorax or hemothorax, n (%) | 3 (1) | 3 (1) | 0 (0) | 1.00 |
| Infection, n (%) | 13 (2) | 12 (2) | 1 (5) | 0.34 |
| Allergy, n (%) | 1 (0.2) | 1 (0.2) | 0 (0) | 1.00 |
| Other prolonged hospitalization due to the procedure, n (%) | 9 (1) | 8 (1) | 1 (5) | 0.25 |

All values indicate mean ± standard deviation unless otherwise indicated.
