# Supplementary material for: Impact of Immunosuppressive Therapy on Lead Dislodgement After Cardiac Implantable Electronic Device Implantation
Source: Clin Cardiol. 2024 Jun 18;47(6):e24310. doi: 10.1002/clc.24310 (PMC11184469; doi:10.1002/clc.24310)
Supplement: Supplementary file 5 — Supporting information. [file CLC-47-e24310-s001.docx]

**Supplementary Table 4. Predictors of lead dislodgement**

| **Variable** | **Unadjusted analysis** | | **Adjusted by hypertension** | | **Adjusted by lead addition** | | **Adjusted by NSAIDs** | |
| --- | --- | --- | --- | --- | --- | --- | --- | --- |
|  | **OR (95%CI)** | **p** | **OR (95%CI)** | **p** | **OR (95%CI)** | **p** | **OR (95%CI)** | **p** |
| Immunosuppressive therapy | 16 (3.7-66) | < 0.001 | 15 (3.4-65) | < 0.001 | 14 (2.9-63) | 0.001 | 13 (2.8-57) | 0.001 |
| Hypertension | 0.2 (0.04-0.8) | 0.03 | 0.2 (0.04-0.9) | 0.03 | - | - |  |  |
| Lead addition | 13 (3.2-55) | < 0.001 | - | - | 11 (2.5-53) | 0.002 |  |  |
| NSAIDs | 5.3 (1.1-26) | 0.04 |  |  |  |  | 3.1 (0.5-18) | 0.20 |

NSAIDs: Non-steroidal anti-inflammatory drugs, OR: odds ratio, CI: Confidence interval
